# Bridging the phenomenological gap between predictive basic-symptoms and attenuated positive symptoms: a cross-sectional network analysis

**DOI:** 10.1038/s41537-022-00274-4

**Published:** 2022-08-24

**Authors:** Hendrik Müller, Linda T. Betz, Joseph Kambeitz, Peter Falkai, Wolfgang Gaebel, Andreas Heinz, Martin Hellmich, Georg Juckel, Martin Lambert, Andreas Meyer-Lindenberg, Frank Schneider, Michael Wagner, Mathias Zink, Joachim Klosterkötter, Andreas Bechdolf

**Affiliations:** 1 Department of Psychiatry and Psychotherapy, Faculty of Medicine and University Hospital Cologne, Cologne, Germany; 2 Department of Psychiatry and Psychotherapy, University Hospital, LMU Munich, Munich, Germany; 3 Department of Psychiatry and Psychotherapy, Medical Faculty, Heinrich-Heine-University, Düsseldorf, Germany; 4 Department of Psychiatry and Psychotherapy, Charité University Medicine Campus Mitte, Berlin, Germany; 5 Institute of Medical Statistics and Computational Biology, Faculty of Medicine and University Hospital Cologne, University of Cologne, Cologne, Germany; 6 Department of Psychiatry, Psychotherapy, and Preventive Medicine, Ruhr University Bochum, Bochum, Germany; 7 Department of Psychiatry and Psychotherapy, Centre for Psychosocial Medicine, University Medical Centre Hamburg-Eppendorf, Hamburg, Germany; 8 Central Institute of Mental Health, Medical Faculty Mannheim, University of Heidelberg, Heidelberg, Germany; 9 Department of Psychiatry and Psychotherapy, Rhineland Friedrich Wilhelms University of Bonn, Bonn, Germany; 10 District Hospital for Psychiatry, Psychotherapy, and Psychosomatics, Ansbach, Germany; 11 Department of Psychiatry, Psychotherapy, and Psychosomatics, Vivantes Klinikum am Urban and Vivantes Klinikum im Friedrichshain, Academic Hospital Charité University Medicine, Berlin, Germany

**Keywords:** Psychosis, Schizophrenia

## Abstract

Attenuated positive symptoms (APS), transient psychotic-like symptoms (brief, limited intermittent psychotic symptoms, BLIPS), and predictive cognitive-perceptive basic-symptoms (BS) criteria can help identify a help-seeking population of young people at clinical high-risk of a first episode psychosis (CHRp). Phenomenological, there are substantial differences between BS and APS or BLIPS. BS do not feature psychotic content as delusion or hallucinations, and reality testing is preserved. One fundamental problem in the psychopathology of CHRp is to understand how the non-psychotic BS are related to APS. To explore the interrelationship of APS and predictive BS, we fitted a network analysis to a dataset of 231 patients at CHRp, aged 24.4 years (SD = 5.3) with 65% male. Particular emphasis was placed on points of interaction (bridge symptoms) between the two criteria sets. The BS ‘unstable ideas of reference’ and “inability to discriminate between imagination and reality” interacted with attenuated delusional ideation. Perceptual BS were linked to perceptual APS. Albeit central for the network, predictive cognitive basic BS were relatively isolated from APS. Our analysis provides empirical support for existing theoretical accounts that interaction between the distinct phenomenological domains of BS and APS is characterized by impairments in source monitoring and perspective-taking. Identifying bridge symptoms between the symptom domains holds the potential to empirically advance the etiological understanding of psychosis and pave the way for tailored clinical interventions.

## Introduction

In the majority of the cases, the onset of the first psychotic episode is preceded by a prodromal phase with a duration of several years^1^. Initially, predominantly unspecific changes in mood, such as anxiety, irritability, depression, and social withdrawal, are reported^2,3^.

Predictive risk symptoms typically emerge later in the prodromal phase. Two prospective evaluated symptom sets are defined: 1. the ultra-high risk criteria and 2. the basic symptoms criteria^4,5^. The UHR criteria include three subgroups: 1. brief, limited intermittent transient psychotic symptoms (BLIPS), 2. attenuated positive symptoms (APS), and 3. a subgroup defined by genetic risk and/or schizotypal disorder with substantial functional decline^6^.

From a clinical point of view, the APS are of the utmost importance, as they occur most frequently^4,7^. The content of APS is similar to full-blown psychotic symptoms. However, APS are less severe and more transient than full-blown psychotic symptoms, and the ability of a person to reflect upon their symptoms as potential signs of a disorder is relatively maintained^8^. BLIPS are defined by the presence of at least one of the following symptoms: hallucinations, delusions, formal thought disorder for less than seven days resolving spontaneously, whereby reality testing is not maintained.

Basic symptoms (BS), on the other hand, do not include psychotic symptoms such as delusions or hallucinations, and reality testing is preserved^9^. BS are disorders of drive, affect, thought, speech processes, perception, proprioception, and motor functions^9^ and are assumed to express the basic (self) disorder of psychosis. Thus, BS should be present throughout the whole course of psychosis^9^. By definition, BS are subjective experiences and do not have to be observable or objectifiable^9^. However, subjectivity is not synonymous with moderate symptom severity, as BS are often associated with a high level of psychological distress^10^. From Huber’s comprehensive set of BS, a cluster of cognitive disturbances BS (COGDIS) and a cluster of cognitive-perceptive BS (COPER, see Table 1) proved to be particularly predictive for a first episode psychosis in help-seeking people at clinical high-risk^4,11^.

**Table 1:** Complete list of predictive basic symptoms illustrated by typical statements from patients.

SPI-A Item No.	Basic symptom name	Typical statement^a^
B1	Inability to divide attention^b^	I can’t focus on driving and listening to the radio simultaneously. I have to concentrate on one or the other.
C2	Thought interferences^b, c^	When I try to focus, inappropriate words come to my mind and distract me.^d^
C3	Thought blockages^b,c^	Whenever I want to think about something, I cannot think. No thoughts come; my head remains empty.
C4	Disturbance of receptive speech^b,c^	It happens that I suddenly can no longer grasp the most straightforward words.^a^
C5	Disturbance of expressive speech^b^	Often speaking doesn’t work correctly, although I have the words I want to say in my mind.
D3	Thought pressure^b,c^	There are just too many thoughts. It’s like ten different pieces of music playing at the same time, and you cannot tell one from the other.
D4	Unstable ideas of reference, “subject-centrism”^b,c^	When strangers are laughing in the street, it strikes me as they are laughing at me. Then I quickly discard this thought.
		When I was listening to the radio, the idea that the lyrics had some special meaning intended for me suddenly popped up in my head. Of course, I knew straight away that it was just my imagination, a kind of weird thing. I did not have to think twice about it to know that.
O1	Thought perseveration^c^	When I am having a conversation, I have to think about recent conversations about people and things that I don’t want to think of.
O2	Decreased ability to discriminate between ideas and perception, fantasy and true memories^c^	Occasionally, when I see something, I’m unsure if it is real or only in my imagination.
		I thought of my grandparents. Then a weird thing happened: I couldn’t remember if I knew my grandparents properly, if they were real or if they were just in my imagination. Did I know them, or had I made them up?
O3	Disturbances of abstract thinking^b^	I must stick to the facts. It is difficult for me to understand metaphors.
O4 (ten subitems)	Other visual perception disturbances^c^	Sometimes things looked distorted or warped.
		Occasionally, everything looked like it had moved far away.
		Often, I don’t grasp the whole picture; then, I see only parts, e.g., faces or objects.
O5 (two subitems)	Other acoustic perception disturbances^c^	I often hear undefined noises such as knocking, hissing, or buzzing.
O7	Captivation of attention by details of the visual field^b^	I cannot just look through a window without cracks, smudges, etc., attracting my attention to such an extent that it disturbs me.
O8	Derealization^c^	I had a feeling of unreality. As if everything was an imitation of reality - similar to a staged theater set.

^a^The typical statements in Table 1 are taken literally or in a modified form from the Bonn Scale for the Assessment of Basic Symptoms (BSABS) and the Schizophrenia Proneness Instrument, Adult Version (SPI-A) manuals, as well as from the article of Eisner et al., 2017 (Eisner et al. 2018), and transcribed diagnostic interviews from the first author (HM) within the CHR service at the university hospital of Cologne, Germany.

^b^COGDIS.

^c^COPER.

^d^Affectively neutral words and other cognitions without a negative connotation.

Based on UHR and/or predictive BS criteria, clinicians can define a help-seeking population of primarily young people at clinical high-risk for a first episode psychosis (CHRp). The operational definition of the CHRp mental state allows to prospectively identify people with an incipient risk of transition to psychosis of up to 20% within 24 months and 22% at 36 months^5^.

Following this line of thought, it has been suggested that BS could progress, via the intermediate stage of APS and/or BLIPS, into full-blown positive symptoms^12–15^. The progression from BS to psychotic symptoms does not imply that BS disappear in the acute stage of psychosis. Rather, it can be said that positive symptoms superimpose BS^12^. Indeed, Huber conceptualized BS as psychotic symptoms “in the making.”^15^ The links between the two symptom sets are of particular interest as these links may help to gain insight into psychotic symptoms^12–14,16^.

We are aware of two studies relating BS to attenuated positive symptoms on an individual symptom level. Klosterkötter based his longitudinal analysis on symptom frequencies in patients with predominantly paranoid-hallucinatory schizophrenia. Klosterkötter found evidence for transition sequences from BS through the intermediate phenomena of derealization and depersonalization, to first-rank symptoms *sensu* Schneider^12,17^. The evidence for the transition from BS to first rank symptoms was later incorporated into a phenomenological model of the transition from self-disorders to ego-disorders^16^.

The most recent study on the symptom level is the cross-sectional network-analysis of at-risk and psychotic symptoms by Jimeno et al.^14^ identified the BS *thought pressure*, *thought interferences*, *hypersensitivity to sound/noises, and changed intensity/quality of acoustic stimuli* as the links to positive symptoms and cognitive-disorganized clusters in their network. Jimeno et al. adopted a broader sampling approach, including help-seeking adults at CHRp (*n* = 203), adults with first-episode psychosis (*n* = 153), or adults with major depression (*n* = 104) in their analysis. Their sampling approach comes with the advantage that the results may be more generalizable to the population of help-seeking young adults in CHR services. However, the inclusion of first-episode psychosis and major depression samples may have obscured important relationships between BS and APS in their CHRp sample.

We conclude that there is some empirical evidence for an association between BS and APS. However, empirical studies on the interaction of BS and APS and to support phenomenological accounts of psychosis^16^ are still scarce. Given the lack of knowledge in the interaction of non-psychotic BS and psychotic symptoms, we might refer to this lack of knowledge as the phenomenological gap. We believe that after the pioneering studies, which included a broader spectrum of mental disorders and full-blown psychosis, the next step is an analysis confined to a CHRp sample. A CHRp sample is an essential precondition for the investigation of the associations between BS and attenuated positive symptoms, as possible associations may be altered or obscured by samples from other populations.

Recently, essential insights into the relationship between psychopathological symptoms have been made within a framework termed the network approach to psychopathology^18–20^. At the heart of the network approach lies the conceptualization of symptoms as distinct components that can influence, maintain, and interact with other symptoms^18,21^. Thereby, the network approach replaces the idea that symptoms are implicitly reflective of specific psychiatric disorders with the notion that symptoms are active, causal ingredients of the disorder itself^21^. In other words, there is no latent factor assumed to which psychopathological symptoms are traced back; instead, the interaction between symptoms is believed to contribute to mental disorders. E.g., suspiciousness may be associated with social withdrawal. However, social withdrawal may also be correlated to developing depressive symptoms. Methodological, a network-based approach to psychopathology allows a data-driven, weighted identification of pathways between different symptom sets. Points of interaction between different symptom sets are termed bridge symptoms. Bridge symptoms are particularly suitable for our analysis of the association of BS and attenuated positive symptoms, as they define important nodes of interaction in a network.

## Aims of the study

Our analysis aims to identify interactions between BS and APS (i.e. bridge symptoms) as an empirically-based understanding of the factors that may influence interactions between these symptom sets are not well-established. Interpreting the interrelationships between the different symptom domains of BS and APS is the primary aim of this analysis and may help elucidate the phenomenological gap between BS and APS. Thus, we aim to increase knowledge concerning the connection (bridge symptoms) between nonpsychotic BS and (pre)psychotic positive symptoms.

## Results

### Sample

After excluding subjects with more than 50% missing values in the network variables of interest (*n* = 1), the final sample comprised *n* = 231 subjects; the demographic and clinical characteristics of the sample are summarised in Table 2. For better readability, referring to APS includes the n = 13 cases that met the BLIPS criterion

**Table 2:** Demographic and clinical characteristics of the sample.

Variable	PREVENT sample (*N* = 231)
Age (years)	24.5 (5.3)
Sex (% male)	64.7
SIPS	
Positive	7.2 (4.3)
Negative	10.5 (5.7)
Disorganization	3.6 (2.5)
General	7.8 (3.6)
MADRS sum	19.8 (7.8)
SOFAS current	52.8 (12.6)
CHR criteria	
COGDIS (%)	18.5
APS (%)	71.4
BLIPS (%)	5.6
GRFD	8.2
Missing values (%)	<1

Descriptive statistics represent mean (SD) unless otherwise stated.

*CHR* clinical high risk, *GRFD* genetic risk and/or schizotypal disorder and substantial functional decline, *MADRS* Montgomery–Åsberg Depression Rating Scale, *SIPS* structured Interview for psychosis-risk syndromes, *SOFAS* social and occupational functioning assessment scale.

### Network stability

**Fig. 1:**
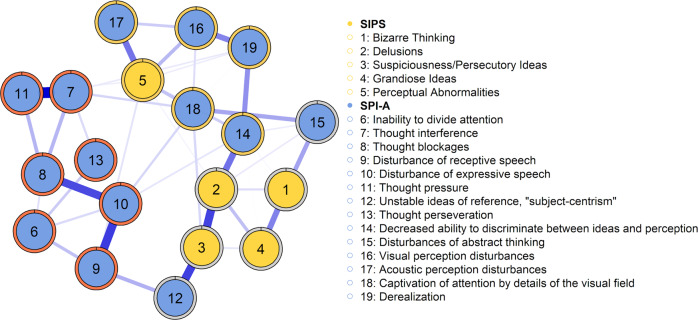
Network of the 19 SIPS and SPI-A items for CHR participants (*n* = 231). Blue lines represent positive associations between two nodes. The wider and more saturated the edge, the stronger the association. We computed a force-directed layout for the visualization of the network. Edges with weights smaller than .05 were omitted from the graph. Node coloring reflects the predefined network communities (yellow: SIPS-P items; blue: SPI-A items). Coloring each node’s border reflects the three communities (clusters) detected with the walktrap algorithm.

Bootstrapping edge weights showed that the smaller edges in the network were prone to sampling variation, as could be expected given the sample size. However, the strongest edges in the network were significantly different from zero and almost always included across all bootstrapped networks. Accordingly, we only present and interpret the strongest edges between bridge symptoms in the network. These are the edges between items 3 and 12, 2 and 14, as well as 5 and 17 (see Fig. 1). The edges of items 3 and 12 and the edge of items 5- and 17 were included in all bootstrapped networks. The edge of items 2 and 14 was not included in 0.01% of the bootstrapped networks. The stability of bridge strength centrality and edge connections were high in the generated network (both CS = 0.52). Stability of strength centrality was adequate (CS = 0.34). This means that the correlation of the results after dropping a substantial number of participants remained quite high, suggesting that the centrality estimates in the original network could be considered stable. Figures of the robustness analysis are available in the supplementary materials.

### Network analysis

The estimated symptom network is shown in Fig. 1. Of the possible 171 edges between the 19 symptoms, 71 were retained. Within the SIPS-P community, the strongest associations were found between *suspiciousness/persecutory ideas* (3) and *delusions* (2). Within the SPI-A community, the strongest associations were the edge between *thought interference* (7) and *thought pressure* (11), as well as the edge between *thought blockages* (8) and *disturbances of receptive speech* (10). Among the most robust connections between items from the two different communities was the edge between *unstable ideas of reference*(^5^) and *suspiciousness/persecutory ideas* (3)*;* the edge between *decreased ability to discriminate between ideas and perception* (14) and *delusions* (2)*;* as well as the edge between *acoustic perception disturbances* (17) and *perceptual abnormalities* (5).

**Fig. 2:**
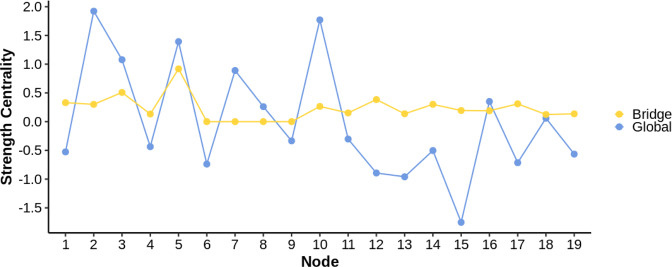
Global and bridge strength centrality (z-standardized) for each node included in the network. Blue dots and lines denote the centrality of the nodes for the overall network. Yellow dots and lines denote the bridge centrality, i.e., those nodes in the network that facilitate the flow of information between the attenuated positive symptoms and predictive basic symptoms. Higher values indicate higher centrality strength. For a legend of the individual symptom labels (1–19), see Fig. 1.

Figure 2 presents centrality plots for the nodes in the network. Analyzing global strength centrality (i.e. irrespective of predefined communities), among the most central nodes were *delusions* (2), *disturbances of expressive speech* (10), *perceptual abnormalities* (5), and *suspiciousness/persecutory ideas* (3). In terms of bridge strength centrality (i.e., taking into account the predefined communities of SIPS and SPI-A items), the nodes that showed the most inter-community connectivity were *perceptual abnormalities* (5), *suspiciousness/persecutory ideas* (3), *unstable ideas of reference*(^5^), and *bizarre thinking* (1).

## Discussion

To our knowledge, this is the first network analysis of attenuated positive symptoms and the predictive basic symptoms (BS) confined to a CHRp sample. Less attention has been paid to the interactions between APS and other symptom sets that contribute to the CHRp state. The simultaneous analysis while controlling for all other associations in the network allows for weighting and helps identify critical points of interaction between basic symptoms and attenuated positive symptoms. The results identified three strong links between BS and attenuated positive symptoms.

The first bridge symptom suggests that not distinguishing *between ideas and perception or fantasy and true memories and objects* interacts with delusional thinking. Schultze-Lutter et al. (2007) give a telling clinical illustration of this basic symptom: “In the last weeks, my thoughts became stronger and stronger. Sometimes I could not tell if I was imagining something or if it was real”^22^. This inability to discriminate between imagination and reality shows considerable overlap to the source monitoring deficit as formulated by Johnson (1997): “For example, people sometimes confuse what they inferred or imagined and what actually happened, what they saw and what was suggested to them, one person’s actions and another’s, what they heard and what they previously knew, and fiction and fact”^23^. Thus, we interpret the predictive BS inability to discriminate between imagination and reality as a phenomenon closely related to the source monitoring deficit. Source monitoring deficits are an essential building block of recent cognitive models on psychotic symptoms^16,24^. Phenomenological authors argued that the distinction between fiction and facts is ensured by the “as if” mode, that is, the ability to evoke memories, imaginations, and the like while distinguishing them from external experience by the reservation of the “as if”^12,16^. The network bridge *between ideas and perception or fantasy and true memories and objects* to *delusional thinking* provides empirical support for the phenomenological notion that a pivotal step in the interaction of attenuated delusion and BS lies in the breakdown of the “as if” mode^12,16^. However, even if our network analysis controls all other variables in the network, delusional ideas and source monitoring deficits could be mediated by a third factor not included in our analysis.

The second bridge from our network analysis confirms a close interaction between *unstable ideas of reference* and *persecutory ideas*^22,25^. A typical statement for *unstable ideas of reference* is: “I have the feeling as if everything is related to me. However, this cannot be true.” (see Table 1). Thus, this symptom is associated with the feeling of being at the center of the events. A fleeting delusional quality is evident in this BS, which the subject can still reject. However, the examples show that the rejection of the feeling of subject-centrism can only be obtained by deliberate cognitive effort. According to phenomenological approaches, reality testing corresponds to the ability to change one’s subjective perspective. Thus, phenomenological accounts highlight the breakdown of the capability “to achieve an exchange of reference frames or perspectives, i.e., to consider the situation—even if only temporarily—with the eyes of the other(s).” [Klaus Conrad, translation by Mishara^26^]. Moreover, our findings are in line with an analysis of symptom sequences in the schizophrenia prodrome that confirms the proximity between *unstable ideas of reference* and *persecutory ideas* found in our analysis^25^.

A content-based comparison of the third bridge from *perceptual abnormalities* (SPI-A) to *acoustic perception disturbances* (SIPS) shows overlaps in the area of low-level acoustic changes (e.g., *acoasm*). Therefore, the strong link demonstrated by our analysis could, in part, be explained by this overlapping content. However, there are also apparent phenomenological differences separating perceptual BS from symptoms closer to the psychosis threshold, such as *thoughts aloud* (*Gedankenlautwerden*) and *attenuated acoustic hallucinations*. We, therefore, assume that basal auditory abnormalities interact with auditory anomalies at a higher level. This finding is in good agreement with the results from Jimeno et al. (2020) who also identified subtle acoustic changes as links to the positive symptoms in their network^14^.

Apart from this similarity, the results of our analysis also differ from the only comparable network analysis in the field^14^. Jimeno et al. (2020) identified the BS *thought pressure* and *thought interferences* as bridge symptoms to *attenuated positive symptoms* and *disorganization*^14^. The differences to Jimeno et al. may reflect the different item selection strategies of the two studies. Jimeno et al. included all symptoms listed in the SPI-A, SIPS, and PANSS in their analysis. For the sake of sufficient statistical power, we restricted ourselves to the predictive BS of the COGDIS and COPER cluster, and we did not include the factor *disorganization* in our analysis.

### Strengths and limitations

Our fine-grained analysis at the symptom level reveals connections that would be lost if we used sum scores of the psychopathological domains. However, *delusional ideas* were measured by SIPS delusion (P1), which is a potpourri of various symptoms such as *perplexity* and *delusional mood, overvalued ideas*, *first-rank symptoms*, *delusional ideas*, and the like. This admixture may have led to obscure correlations in the network. Based on previous findings^12^, it can be assumed that there might be closer associations between the cognitive BS and first-rank symptoms. Symptom-specific instruments for both first-rank and delusional symptoms may be necessary to accurately assess the association between predictive cognitive BS and attenuated positive symptoms.

Another strength is our dataset^27^, which constitutes the largest at-risk sample in analysing links between BS and attenuated positive symptoms (see review in the introduction) with an excellent risk enrichment compared to other recent studies in the field^27^. The data used for this network analysis stems from subjects who consented to a clinical trial (PREVENT); thus, the results may not be generalizable to other help-seeking samples. However, Bechdolf et al. (2011) found no differences between individuals who consented to PREVENT and those who did not^27^.

A further limitation is that our conclusions are based on cross-sectional data. When analysing a link between two nodes in the network analysis, the associations to all other nodes in the network are statistically controlled. Thus, the connection between the BS and attenuated positive symptoms within the set of selected symptoms is potentially indicative of a causal relationship. Research on the progression from BS and attenuated positive symptoms in a retrospective study in first-episode psychosis (*n* = 126)^13^ provide indications of the sequence from BS to APS to psychotic symptoms, albeit not always consistently. The best evidence that BS precede acute psychosis comes from meta-analyses of prospective studies demonstrating that COGDIS and COPER’s BS clusters were predictive of the dichotomized outcome *transition to psychosis* after 6–48 months^4^. Ultimately, only longitudinal studies will be able to make sound conclusions regarding the order of this symptom sequence.

The main finding from our network analysis is that both BS, which form the bridge to delusional ideation, share impaired reality testing as a common denominator. Therefore, the results of our network analysis offer empirical evidence for phenomenological accounts highlighting impairments in perspective-taking and breakdown of the “as if” mode for the aetiology of first psychotic symptoms, stating that: “This is the hallmark of delusion and the reason for its incorrigibility: taking another’s perspective and, thus, a distance from oneself has become impossible”^16^.

The bridge symptoms between non-psychotic and psychotic symptoms identified by our network might pave the way for targeted preventive interventions in CHRp states. Promising preventive interventions include psychoeducation about source monitoring, generating alternative explanations to delusional beliefs^28^, and fostering perspective-taking^29,30^. The feasibility of such interventions within a cognitive behavioral therapy framework has already been demonstrated in a randomized controlled trial in CHRp patients^31^. However, translating our findings into targeted interventions would require a demonstration that our results at the group level can also be replicated at an individual level. Therefore, we propose longitudinal studies of predictive BS and attenuated positive symptoms at an individual level as a next step. Such a longitudinal network analysis study could determine the direction of activation from BS to other psychopathological domains and might inform individualized psychotherapeutic case formulations.

## Methods

### Network analysis

Network analysis allows for quantification and depiction of the strength by which symptoms react with and influence each other, as well as those symptoms that are most central to a disorder^19,32^. In a psychopathological network model, symptoms are depicted as individual nodes connected by edges that reflect the strength and direction of the relationship between pairs of symptoms. *Central symptoms* are nodes that show many strong connections to other symptoms in the network, facilitating the flow of information between other, disconnected nodes^33^. Those symptoms in a network that connect two or more *communities*, i.e. groups of symptoms, can be identified with a relatively new measure called *bridge centrality*^34,35^.

### Participants and Study Design

We used the cross-sectional baseline data from CHRp participants (*n* = 232) recruited within the multicenter *Secondary PREVENTion of schizophrenia: a randomized controlled trial* (PREVENT) (ISRCTN identifier 02658871). The trial protocol was reviewed and approved by the Ethics Committee of the Medical Faculty of the University of Cologne. All participants provided written informed consent before any research activity. Full details on the PREVENT trial are available elsewhere^27^. The CHRp state in PREVENT was defined by APS, BLIPS, COGDIS, and family risk plus reduced functioning as assessed by the Structured Interview for Prodromal Symptoms (SIPS/SOPS)^36^ and the Schizophrenia Prediction Instrument- Adult Version (SPI-A)^22^.

The SIPS/SOPS was designed to rate the current severity of the psychosis-risk symptoms. Positive Symptoms are rated on the SOPS scale that ranges from 0 (absent) to 6 (severe and psychotic). The SPI-A was developed to assess the presence of BS. The SPI-A measures BS on a scale from 0 (absent) to 6 (extreme).

The same interviewer rated the SIPS/SOPS and SPI-A in the respective study center. The intraclass correlations of the masked assessor’s ratings ranged from good (0.69) to excellent (0.98)^37^.

We also report depression scores and level of psychosocial functioning as measured by the Montgomery-Åsberg Depression Rating Scale (MADRS) and the Social and Occupational Functioning Assessment Scale (SOFAS).

### Selection of network items

Overall, we selected 19 items for the present analysis. No other variables were included in the analysis. Five attenuated positive symptoms from the SIPS/SOPS: *Unusual thought content/ delusional ideas* (SIPS item P1) *suspiciousness/ persecutory ideas* (SIPS item P2); *grandiosity* (SIPS item P3); *perceptual abnormalities/ hallucinations* (SIPS item P4); and *bizarre thinking* (SIPS item D2). Factor analyses have repeatedly shown that the symptom ‘disorganized thinking’ does not load on the factor ‘positive symptoms’, whereas the symptom ‘bizarre thinking’ belongs to the positive symptoms^38–41^. Given these results, we decided to group the positive symptoms following this evidence.

As laid out above, mainly the BS clusters COPER and COGDIS are predictive in the transition to psychosis (all symptoms from both clusters are reported in Table 1). Hence, it can be assumed that these predictive BS are closely related to the attenuated positive symptoms. Evidence from a meta-analysis suggests that the COGDIS cluster indicates a high shorter-term risk for psychosis (≥1 year)^4^. On the other hand, the COPER cluster may predict transition to psychosis in the longer term (>5 years)^4^. To account for both short-term and long-term risk, we include all symptoms of the COGDIS, as well as the COPER cluster in our analysis. To present a well-powered analysis, we excluded all unspecific BS from the analysis.

### Data analysis

#### Network estimation

We computed and visualized Gaussian Graphical Models (GGM) in the form of regularised partial correlation networks, including the combined nineteen SIPS and SPI-A items^35^. In the resulting network, each *node* corresponds to one of the included SIPS or SPI-A items. A so-called *edge* between two nodes reflects a partial correlation (or, equivalently, conditional dependence relation) between these nodes, i.e., the strength of the association between two items after controlling for all other variables in the network^18^. We recovered the optimal network by minimizing the extended Bayesian Information Criterion (EBIC) within the glasso algorithm^42,43^. The EBIC regularisation parameter γ was set at 0.25 for the analysis, balancing the stability and sensitivity of the analysis^42^. We generated force-directed layouts with the Fruchterman-Reingold algorithm for plotting the networks, which places more strongly connected items at the center of the network^44^. We performed all network estimation procedures using the *R* package *qgraph*^45^.

#### Importance of network items

A common way to assess the importance of a symptom in a psychopathological network is to compute centrality indices, which reflect the connectedness of a given symptom with all other symptoms in the network^35^. We calculated the *strength centrality* of each symptom: a common and stable centrality metric defined as the sum of all absolute values of the associations that a given symptom has with all other symptoms in the network^46,47^.

#### Bridge centrality

Bridge centrality has recently been introduced as a measure to identify those symptoms in a network that facilitate the flow of information between two (or more) communities of symptoms^34^. Communities are groups of predefined symptoms based on theoretical criteria rather than emerging from the network structure. Hence, in contrast to *(global) strength centrality*, *bridge centrality* takes any theoretically defined grouping of symptoms in the network structure into account. This paper focuses on *bridge strength*, which measures a given symptom’s total connectivity with symptoms from the other community by the sum of its absolute inter-community associations. We defined two communities: one community comprising all SPI-A items from the COPER and COGDIS-clusters, and another community including those symptoms pertaining to attenuated positive symptomatology assessed by the SIPS.

#### Communities

We explored the way nodes within the estimated network cluster together using a random walk algorithm (walktrap)^48^ as implemented in the *R* package *igraph*^49^. Nodes that cluster together in communities may be part of the same latent variable or dimension^50^.

#### Network accuracy and stability

As recommended, we performed several follow-up analyses on the calculated networks to assess their robustness using the *R* package *bootnet*^35,51^. These analyses show how accurately the edges in the network are estimated by constructing a 95% confidence interval (CI) around them and indicate how stable centrality is estimated via the centrality-stability (CS) coefficient. This coefficient indicates the maximum proportion of observations that can be dropped while confidently (95%) retaining results that correlate highly (*r* > 0.7) with the results obtained in the original sample. A CS coefficient of 0.25 or above indicates adequate stability, and a coefficient of 0.50 or above indicates high stability^35^. For all analyses, we used 5,000 bootstrap samples.

## Supplementary information

Supplemental Material Mueller et al. Network Analysis

## Data Availability

The data that support the findings of this study are available on upon reasonable request from the first author (H.M.).
