# Supplementary material for: Bridging the phenomenological gap between predictive basic-symptoms and attenuated positive symptoms: a cross-sectional network analysis
Source: Schizophrenia (Heidelb). 2022 Aug 24;8(1):68. doi: 10.1038/s41537-022-00274-4 (PMC9402628; doi:10.1038/s41537-022-00274-4)
Supplement: Supplementary file 1 — Supplemental Material Mueller et al. Network Analysis [file 41537_2022_274_MOESM1_ESM.pptx]

## Slide 1
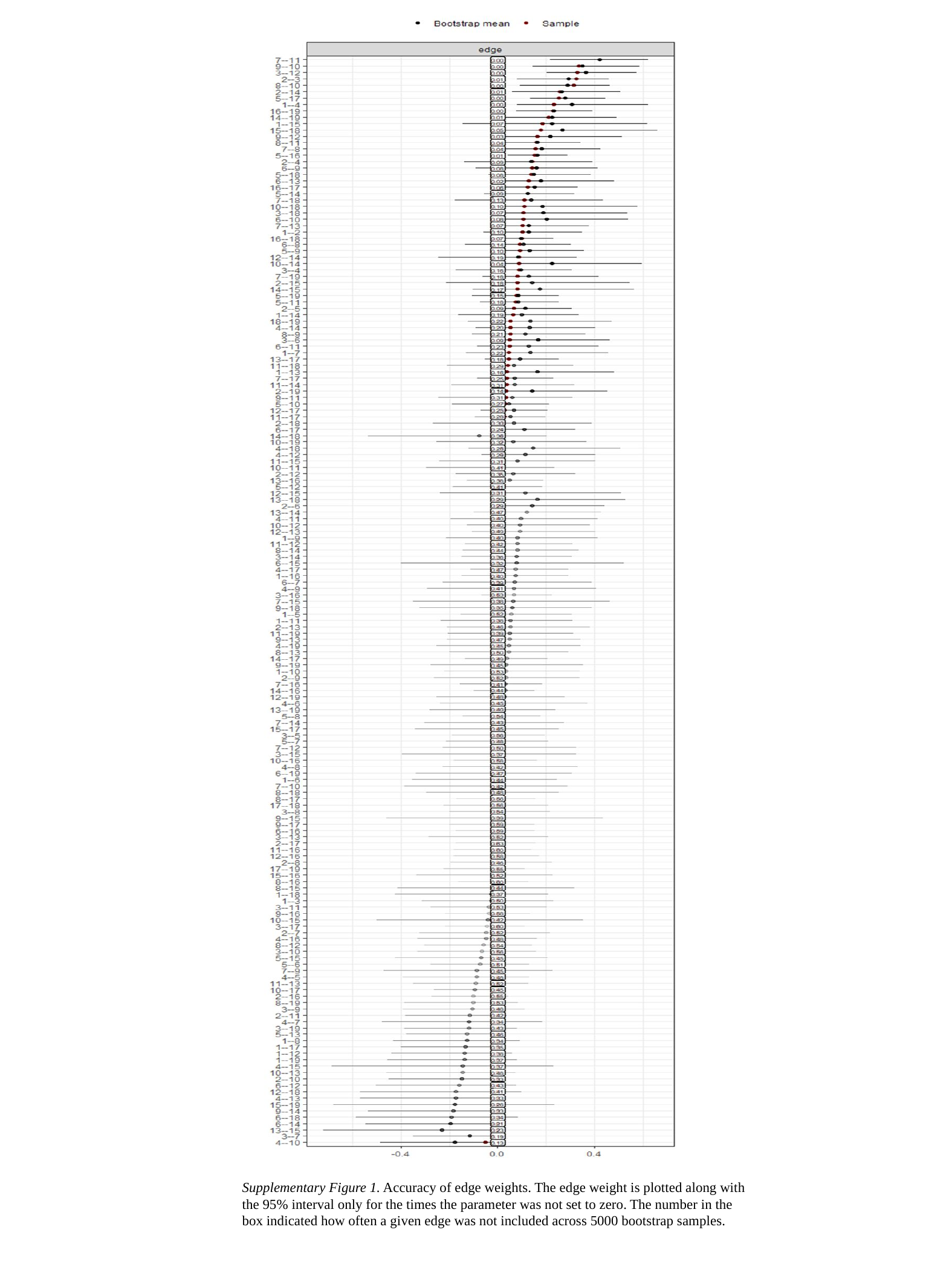

Supplementary Figure 1. Accuracy of edge weights. The edge weight is plotted along with the 95% interval only for the times the parameter was not set to zero. The number in the box indicated how often a given edge was not included across 5000 bootstrap samples.

## Slide 2
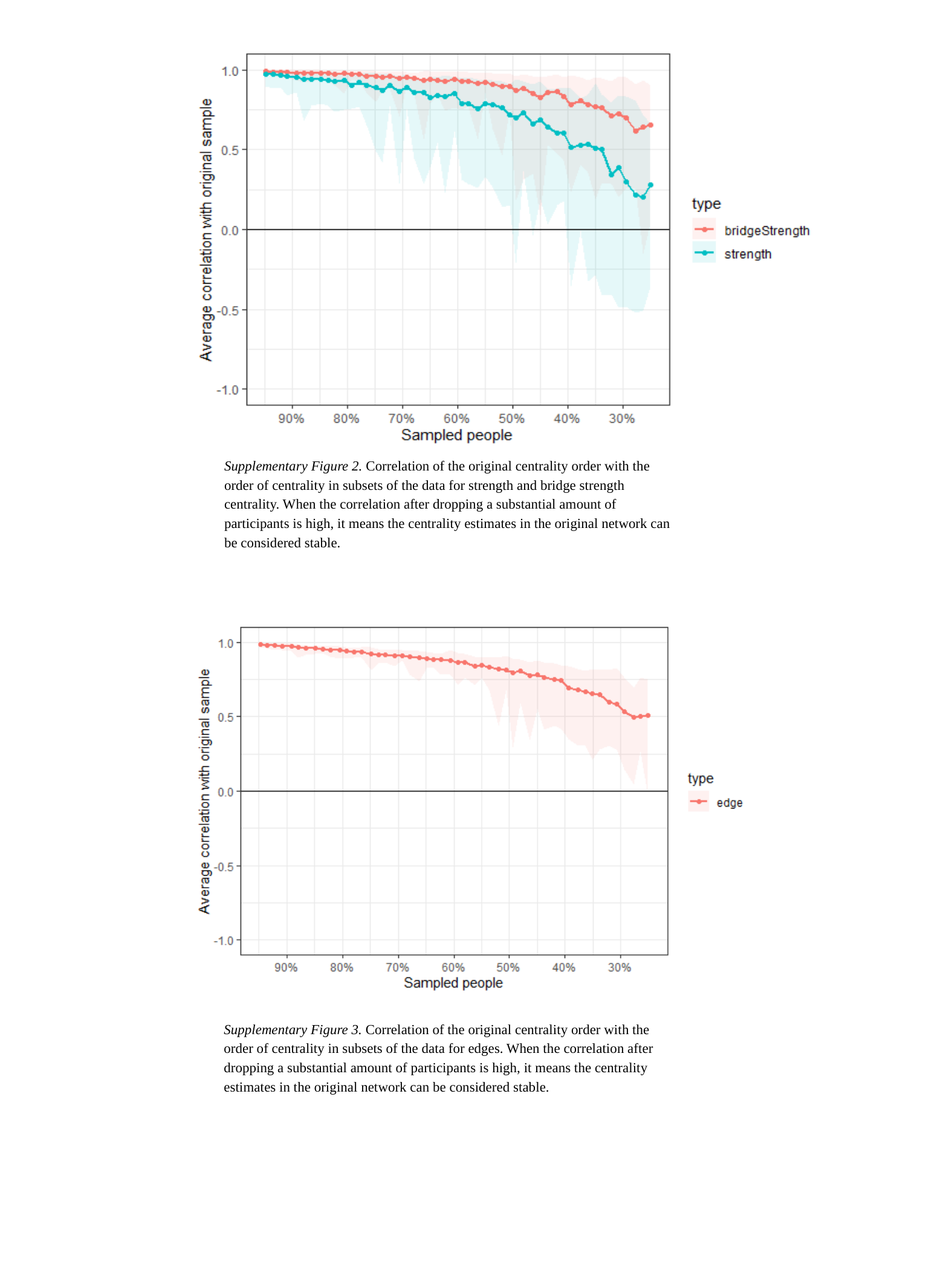

Supplementary Figure 2. Correlation of the original centrality order with the order of centrality in subsets of the data for strength and bridge strength centrality. When the correlation after dropping a substantial amount of participants is high, it means the centrality estimates in the original network can be considered stable.
Supplementary Figure 3. Correlation of the original centrality order with the order of centrality in subsets of the data for edges. When the correlation after dropping a substantial amount of participants is high, it means the centrality estimates in the original network can be considered stable.
